# Investigation of dual modification on physicochemical, morphological, thermal, pasting, and retrogradation characteristics of sago starch

**DOI:** 10.1002/fsn3.2837

**Published:** 2022-03-24

**Authors:** Pantea Ghalambor, Gholamhassan Asadi, Abdorreza Mohammadi Nafchi, Seyed Mahdi Seyedin Ardebili

**Affiliations:** ^1^ Department of Food Science and Technology, Science and Research Branch Islamic Azad University Tehran Iran; ^2^ Department of Food Science and Technology, Damghan Branch Islamic Azad University Damghan Iran; ^3^ Food Technology Division School of Industrial Technology Universiti Sains Malaysia Penang Malaysia

**Keywords:** acid hydrolysis, frozen dough, hydroxypropylation, retrogradation, syneresis

## Abstract

The aim of this study was to evaluate the characteristics of dually modified sago starch by acid hydrolysis (AH)‐hydroxypropylation (HP). For this purpose, sago starch was modified with the combination by AH (5–20 h hydrolysis times) followed by HP (5%–25% ratio of propylene oxide) processes. The results showed that the dual modification of the sago starch structure didn't have a significant effect on the size of starch granules, and the granule size was in the range of 0.005–0.151 µm; however, the pasting properties and the glass transition temperature decreased significantly (*p* < .05). Increasing the level of propylene oxide from 5% to 25% caused a significant increase in the substitution degree (DS) and swelling ability of starches and reduced the syneresis, while with increasing acid hydrolysis time from 5 h to 20 h, starch swelling decreased and syneresis increased (*p* < .05). AH process at high hydrolysis times (20 h) increased the gelatinization temperatures and decreased retrogradation temperatures. Increasing the level of propylene oxide in both single and dual modification reduced the temperatures and enthalpy of gelatinization and retrogradation of sago starch. In summary, dually modified sago starch has a great potential to use in specific food products such as frozen dough or frozen bakery products.

## Graphical abstract

Evaluation of the characteristics of dually modified sago starch by acid hydrolysis (AH)‐hydroxypropylation (HP). Increasing the level of propylene oxide from 5% to 25% caused a significant increase in the substitution degree (DS) and swelling ability of starches and reduced the syneresis, while with increasing acid hydrolysis time from 5 h to 20 h, starch swelling decreased and solubility in water increased (*p* < .05).

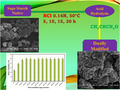

## 1 INTRODUCTION

Starch is a major and noticeable storage polysaccharide in plant sources that consists of two parts, including amylose (the linear polymer) and amylopectin (the highly branched polymer) (Sondari et al., 2021). Starch is an abundant, available, cheap, and biodegradable polysaccharide found in different parts of plants such as fruits, leaves, roots, flowers, seeds, and stems, which it uses as an important source of energy and carbon. Cereals, roots, tubers, legumes, and some unripe and green fruits such as mangos and bananas are major sources of starch. Starches in various plant sources have different and unique sizes, shapes, compositions, and structures (Ashogbon, 2021; Krithika & Ratnamala, 2019).

The sago plant (*Metroxylone sagu* Rottb) is one of the sources of starch in the world (Zailani et al., 2021). This starch contains 27% amylose and 73% amylopectin and is mainly grown and exported in Southeast Asia (Ekramian et al., 2021; Xue Mei et al., 2020). Sago starch in its natural form has several disadvantages such as low solubility in cold water, formation of chewy and opaque paste, spoilage during storage, syneresis, easy retrogradation, lack of emulsifying property, and so on, which limit its use in various food products and food industry (Zainal Abiddin et al., 2018). The modification process is often used to improve the application and functionality of starches, which is performed by physical, chemical, genetic, or enzymatic methods alone or in combination (Hartiningsih et al., 2020).

The chemical modification process of starch structure involves the introduction of different functional groups (such as ester, carboxylic, ether, and amino groups) into the structure of starch molecules without changing the size and shape of molecules, which causes significant changes in the physicochemical and functional properties of starch. For example, modifying the structure of native starches improves their behaviors in terms of retrogradation, pasting, and gelatinization (Hong et al., 2020). The hydroxypropylation process is a chemical modification method in which hydrophilic bulky groups of hydroxyl propylene introduce to the structure of starch, thereby increasing the solubility, cohesiveness, paste clarity, enzymatic digestibility, and freeze‐thaw stability of the starch. Because hydroxypropylated starches have enhanced characteristics, they are used in food products, pharmaceutical capsules, and biodegradable films (Chen et al., 2021). One of the most widely used methods to modify the structure of starch is acid hydrolysis, in which different dilute acids (H_2_SO_4_, HCl, or H_3_PO_3_) are used to treat the starch slurry under temperature lower than the gelatinization temperature (40–60°C) (Li et al., 2020). This chemical method is inexpensive and can change the microstructure, crystalline, viscoelastic, gelatinization, and digestibility characteristics of starch (Aminian et al., 2013).

The dual modification process often improves the functionality of starches better than a single modification. Homogeneous and heterogeneous methods are used for dual modification of starches. In the initial type (homogeneous), two similar methods are used, for example, two physical or two chemical methods. However, in the second type (heterogeneous), two different methods are used, such as combining the physical and chemical methods (Ashogbon, 2021). Previous research has shown that the combined use of hydroxypropylation and acid hydrolysis processes (dual modification) improves the properties and performance of starches, and these chemical processes have a synergistic effect (Fouladi & Mohammadi Nafchi, 2014; Javadian et al., 2021; Li et al., 2018).

So far, various methods such as ozone oxidation (Sumardiono et al., 2021), octenylsuccinylation (Zainal Abiddin et al., 2018), steam explosion followed by acid hydrolysis (Hartiningsih et al., 2020), 3‐chloro‐2‐hydroxypropyl trimethylammonium chloride (CHPTAC) (Naseri et al., 2019), hydroxypropylation followed by crosslinking (Wattanachant et al., 2003), acid treatment in alcohol (Yiu et al., 2008), and alcoholic‐alkaline treatment (Kaur et al., 2011) have been used to modify the structure and functionality of sago starch. This study was conducted to investigate the effect of dual modification with acid hydrolysis and hydroxypropylation process on the physicochemical, thermal, morphological, retrogradation, pasting, and syneresis properties of sago starch.

## 2 MATERIALS AND METHODS

### 2.1 Materials

Sago starch and propylene oxide were purchased from SIM Supply Company Sdn. Bhd. and Sigma Aldrich Company, respectively. Other chemicals used in this research were prepared by Merck Company and were based on analytical grade.

### 2.2 Preparation of dual modified sago starch

To prepare the dual modified sago starch, first acid hydrolysis (AH) was done by hydrochloric acid, and then hydroxypropylation (HP) of the hydrolyzed starch was performed. The sago starch slurry was prepared by mixing starch (400 g; based on dry weight) with a dilute solution of hydrochloric acid (0.14 N) at 50°C to reach a final weight of 1000 g. While stirring (at 200 rpm), the starch slurry was incubated at 50°C for 5, 10, 15, and 20 h to prepare hydrolyzed sago starch with various molecular weights. After that, the pH of starch suspensions was reached 5.5 by the addition of 1% sodium hydroxide. The starch samples were then washed with distilled water and filtered through Whatman No. 4 filter paper. The hydrolyzed sago starches were kept at 40°C overnight in an oven to dry completely (Abdorreza et al., 2012). To adding hydroxypropyl group to hydrolyzed sago starch, the hydrolyzed starch solution (20% w/v) was mixed with sodium sulfate (20% w/v) and stirred. The pH of the starch suspension was reached above 10.5 with the addition of 5% sodium hydroxide. Different proportions (5, 15, and 25%; based on the dry starch weight) of propylene oxide (as etherifying agent) was added to the starch suspension. After that, the starch samples were capped and stirred at 200 rpm for 30 min at room temperature. The pH of starch suspensions was then reached 5.5 using 10% HCl. The dual modified starch samples were washed with distilled water until all sulfate was removed. The modified starches were heated in an oven (at 40°C) until they reached a moisture content of 10%. After the drying process, the starches were ground and then passed through the sieve with meshes of 250 µm (Aminian et al., 2013).

### 2.3 Scanning electron microscopy (*SEM*)

The morphology or microstructure of the sago starch granules was determined using *SEM* (Leica Cambridge). For this purpose, 1 g of starch was coated with a layer of gold/palladium and transferred to the *SEM* and finally observed at 20 kV (Majzoobi et al., 2012).

### 2.4 The molar substitution determination (MS)

100 mg of starch was mixed with 25 ml of H_2_SO_4_ (0.5 M) and then heated at 60°C in a water bath to dissolve the starch sample. The starch solution was cooled at room temperature and diluted with distilled water (to a volume of 100 ml). The starch solution (1 ml) was transferred into graduated test tubes (25 ml) and immersed in an ice bath, and concentrated H_2_SO_4_ (8 ml) was incorporated dropwise. After complete stirring, the tubes were kept for 20 min in a hot water bath and then transferred to an ice bath. 0.6 ml of ninhydrin reagent solution (3% ninhydrin in 5% Na_2_S_2_O_5_) was incorporated into the solutions. After shaking, the tubes were placed in a hot water bath at 25°C for 100 min. The volume of solution was reached 25 ml with the addition of concentrated H_2_SO_4_ and stirred and kept still for 10 min. The absorbance of the starch solution was read at 590 nm. MS of the starch sample was obtained through the following equation: where W and M are the equivalent hydroxypropyl in 100 g of starch, and the molecular weight of C_3_H_6_O, respectively (Liu et al., 2010).

$$\text{MS}=\frac{162W}{100‐\left(M‐1\right)W}$$

### 2.5 The water solubility and swelling determination

1 g of starch was transferred to a plastic centrifuge tube (50 ml) and mixed with distilled water (30 ml). While stirring, the tube was heated in a hot water bath at 95°C for 30 min. The sample was then cooled with cold water to reach room temperature and then centrifuged for 15 min at 700 × g. The resulting supernatant was transferred to a container and dried (at 120°C for 4 h). Finally, the starch sample was weighed, and its water solubility and swelling amounts were obtained through the following equations (Trela et al., 2020):

$$\text{Water}\;\text{solubility}\;\left(\%\right)=\frac{\text{dried supernatant weight}}{\text{dry starch weight}}\times 100$$

$$\text{Swelling}\;\left(g/g\;\text{dried starch}\right)=\frac{\text{sediment weight inside the centrifuge tube}}{\text{dry starch weight}}$$

### 2.6 The pasting characteristics determination

Alternation in the starch slurry viscosity during heating processing was studied with a rapid viscosity analyzer (RVA). The starch sample (4 g) was mixed with distilled water (25 g). The initial desired speed and the speed of the resting stage were 960 rpm and 160 rpm, respectively. At first, the starch sample was kept at 50°C for 1 min, and then its temperature increased rapidly to 95°C (at a speed of 14°C/min) and kept at this temperature for about 5 min. After that, the sample was cooled to 50°C and kept for 2 min at this temperature. In this experiment, pasting temperature, time to reach peak viscosity, peak, and final viscosity were studied (Karim et al., 2008).

### 2.7 The gelatinization properties and glass transition temperature evaluation

The thermal characteristics of the starches were measured using the differential scanning calorimeter (DSC). The starch suspension in distilled water (in a ratio of 3:1 w/w) was prepared and weighed in the steel container of the DSC device and kept for 24 h at room temperature to reach equilibrium. The container containing the starch suspension was then transferred into the DSC device and heated and scanned at 10–115°C. A DSC container without starch suspension was used as a control. The thermal characteristics of starch samples including onset temperature (To), peak temperature (Tp), conclusion temperature (Tc), and gelatinization enthalpy (ΔH) were calculated from the DSC curves (Majzoobi & Beparva, 2013). The glass transition temperature (Tg) was considered as the middle point between the beginning and the end of the curve changes in the heat flow.

### 2.8 The starch retrogradation investigation

To investigate the retrogradation of starch samples, the heated samples in the DSC test were kept in the refrigerator (4 ± 1°C) for 3 weeks, and then their onset temperature (To), peak temperature (Tp), final temperature (Tf), and gelatinization enthalpy (ΔH) were measured by the DSC device (Ruales & Nair, 1994; Yusnita et al., 2017).

### 2.9 The syneresis determination

To determine the syneresis of starch gel, the starch suspension (5% w/v) was prepared and heated for 30 min at 90°C. The starch suspension was cooled in an ice‐water bath to room temperature and then centrifuged at 3200 rpm for 15 min. Finally, the syneresis of starch gel was reported as a percentage of the amount of water separated after centrifugation (Sodhi & Singh, 2005).

### 2.10 Statistical analysis

Statistical analysis of data obtained from the experiments was done using IBM SPSS Statistics 22.0. One‐way analysis of variance (one‐way ANOVA) followed by Duncan multirange post hoc test was used to compare means at *p* < .05 significance level among different samples.

## 3 RESULTS AND DISCUSSION

### 3.1 Morphology and granular size

**FIGURE 1:**
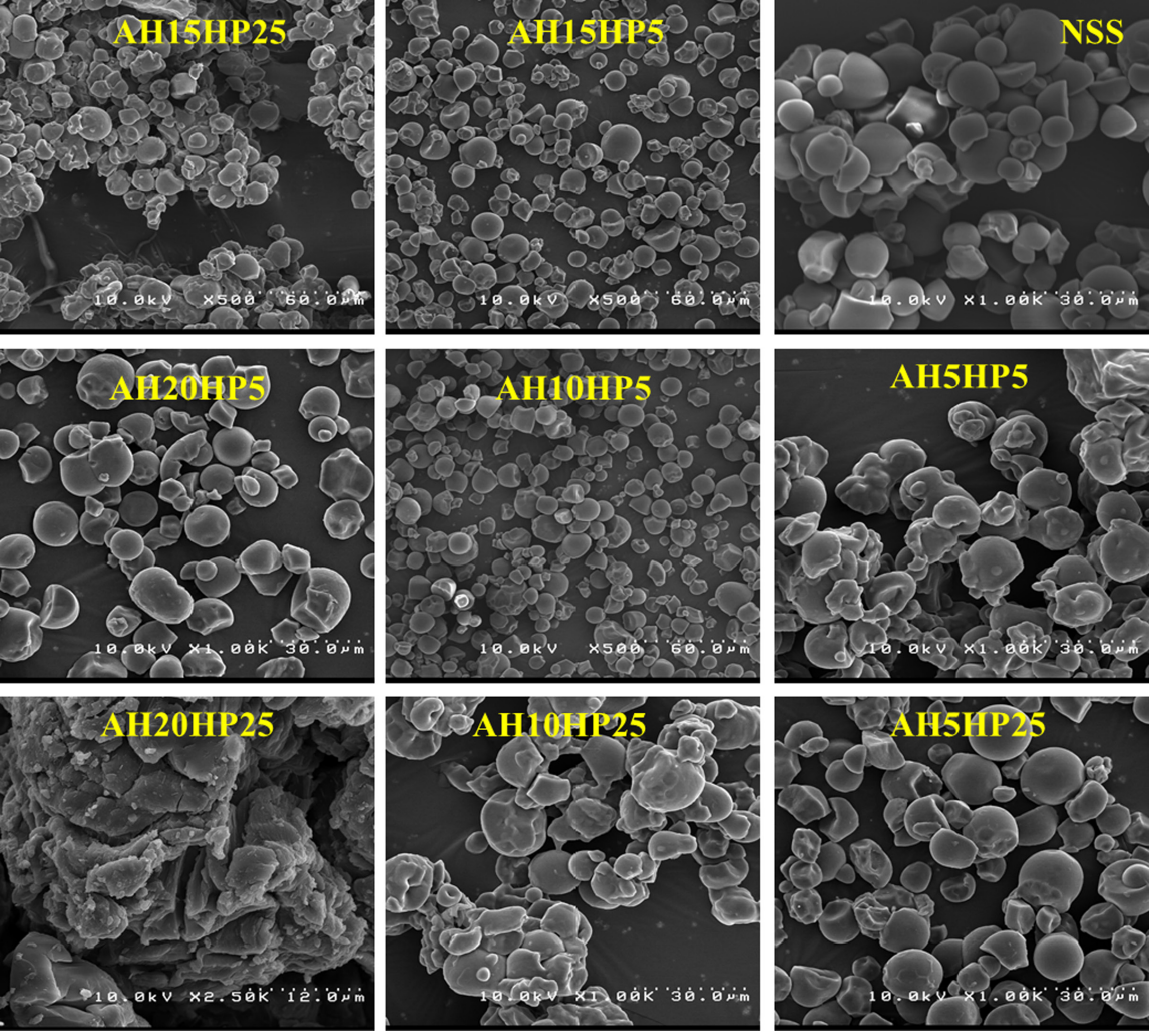
The *SEM* micrographs of the native and dual modified sago starch by acid hydrolysis‐hydroxypropylation. NSS, Native sago starch; The numbers after “HP” represent the propylene oxide ratio (%), and the numbers after “AH” represent the time (h) of acid hydrolysis

Figure 1 shows the *SEM* micrographs of native sago starch and dual modified starches by acid hydrolysis‐hydroxypropylation. As can be seen in the figure, the dual modification of the sago starch structure caused partial destruction of the modified starch structure and increased the depressions in the surface of the granules, and also increased the accumulation of starch granules. Sago starch granules generally had an irregular round shape.

**FIGURE 2:**
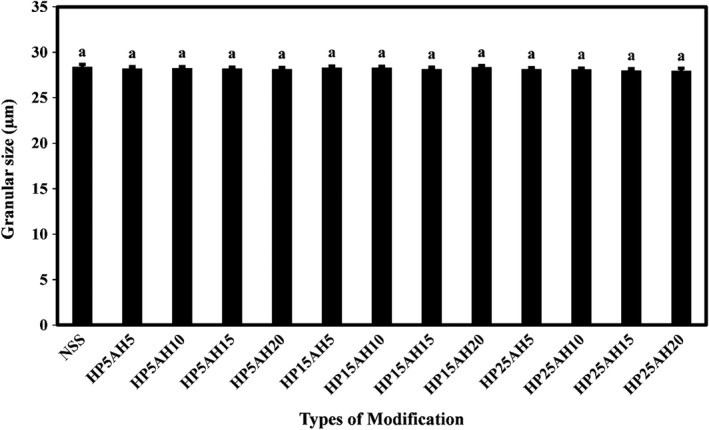
Comparison of granular size values (µm) of native and dually modified sago starch samples by acid hydrolysis‐hydroxypropylation. Bars represent mean (*n* = 3) ± *SD*. Different letters on the bars indicate a significant difference at 5% level of probability among starch samples. NSS, Native sago starch; the numbers after “HP” represent the propylene oxide ratio (%), and the numbers after “AH” represent the time (h) of acid hydrolysis

Granular size is a major factor in the reactions of particles with each other, mixing, and homogenization in food product formulations (Riley et al., 2008). It is generally known that small‐ and medium‐sized starch granules have different applications in the food industry (Omojola et al., 2010). Figure 2 compares the sizes of native and dual modified sago starch granules and shows that the mean size of sago starch granules was in the range of 27.97–28.43 µm, and the dual modification of the starch structure caused a slight reduction in the size of granules; however, this reduction was not significant. Olayinka et al. (2015) observed that native sorghum starch had irregular angular granules, and modification of starch structure by hydroxypropylation and acetylation caused the structure of the modified starches to be destroyed; however, the effect of hydroxypropylation was less. Kaur et al. (2011) found that acid hydrolysis of banana starch increased granule size, but the granule size of sweet potato starch decreased slightly. Tehkhunmag et al. (2008) demonstrated that *SEM* micrographs of hydroxypropylated tapioca starch were approximately identical to those of native starch; however, the dual modification of this starch by carboxymethyl and hydroxypropyl caused the irregular shape of the granules. Surendra Babu et al. (2015) also reported the degradation of sweet potato starch granules due to acid hydrolysis and its increase in time. Hartiningsih et al. (2020) also achieved similar results in the effect of acid hydrolysis on the granular morphology of sago starch.

### 3.2 The molar substitution (MS)

**FIGURE 3:**
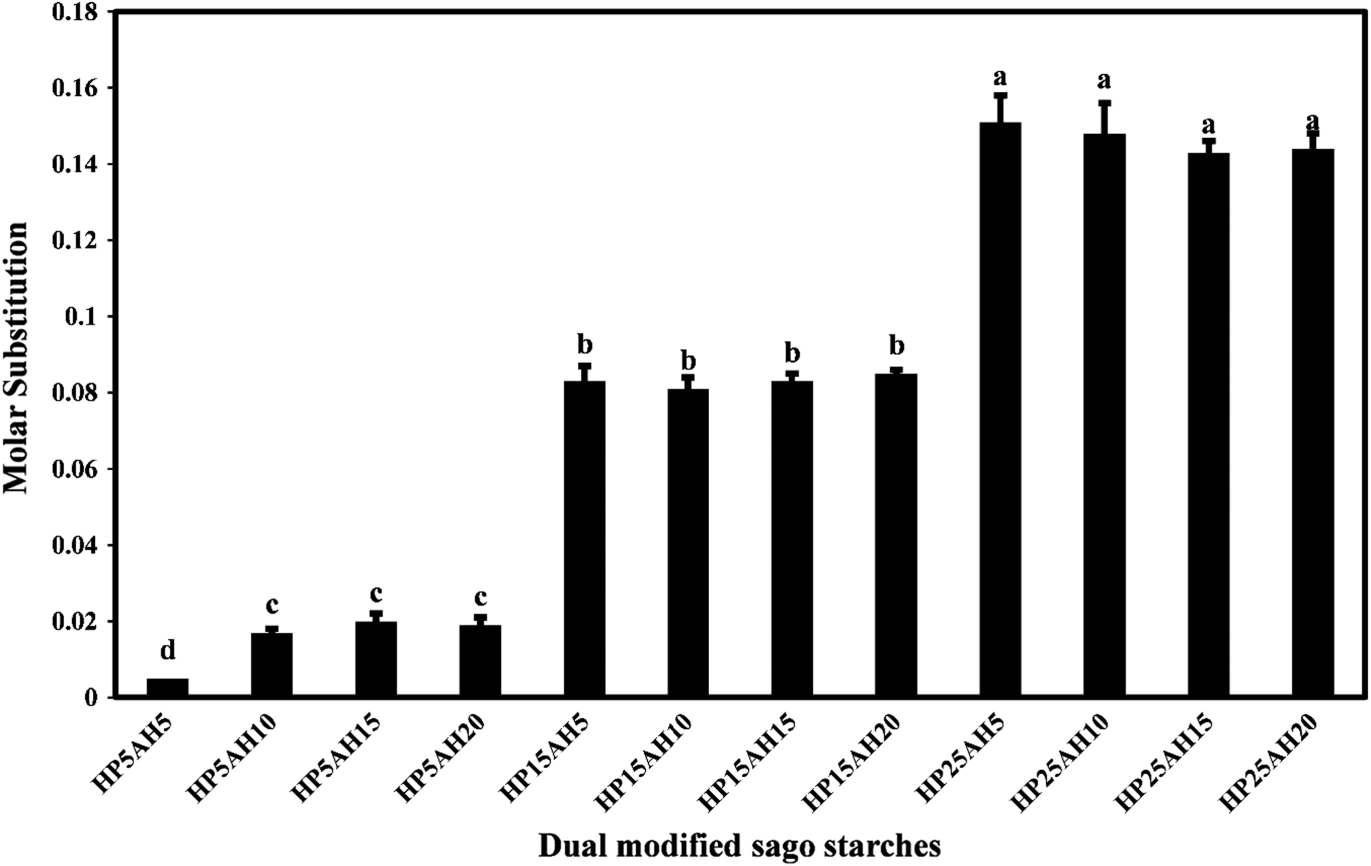
Comparison of MS values of dual modified sago starch samples by acid hydrolysis‐hydroxypropylation. Bars represent mean (*n* = 3) ± *SD*. Different letters on the bars indicate a significant difference at 5% level of probability among starch samples. The numbers after “HP” represent the propylene oxide ratio (%), and the numbers after “AH” represent the time (h) of acid hydrolysis

In this study, the molar substitution (MS) of hydroxypropyl groups in the structure of granular starches was used to determine the efficiency of the hydroxylpropylation and acid hydrolysis process (Arueya & Ojesanmi, 2019). The MS values of dual modified sago starches by acid hydrolysis‐hydroxypropylation are compared in Figure 3. The MS values of starch samples ranged from 0.005 to 0.151. By increasing the propylene oxide ratio from 5% to 25%, the MS of the hydroxyl group in sago starch increased significantly (*p* < .05). Increasing the acid hydrolysis time at a constant level of 5% propylene oxide increased the MS values; however, at other levels of etherifying agent (propylene oxide), increasing the acid hydrolysis time didn't show a significant effect. The efficiency of hydroxypropylation depends on the diffusion or penetration of the etherifying agent and alkaline catalyst intro the granules of starch and the reactivity between the propylene oxide and starch alcoholate nucleophile (Vorwerg et al., 2004). Increasing the level of propylene oxide increases the rate of contact with alkoxide and the active agent near the starch granules (Lawal, 2009). Since the maximum value of MS for modified starches is considered to be 3 (Karim et al., 2008), it can be seen that the modified sago starches produced in this study had a desirable MS value. Arueya and Ojesanmi (2019) by examining the effect of different levels of propylene oxide (4%–12%) on the MS of sweet potato starch, showed that with increasing the ratio of propylene oxide, the MS significantly increased. Other researchers have found similar results (Chen et al., 2021; Choi & Kerr, 2003; Li et al., 2018; Rutkaitė et al., 2016).

### 3.3 Water solubility and water uptake

**FIGURE 4:**
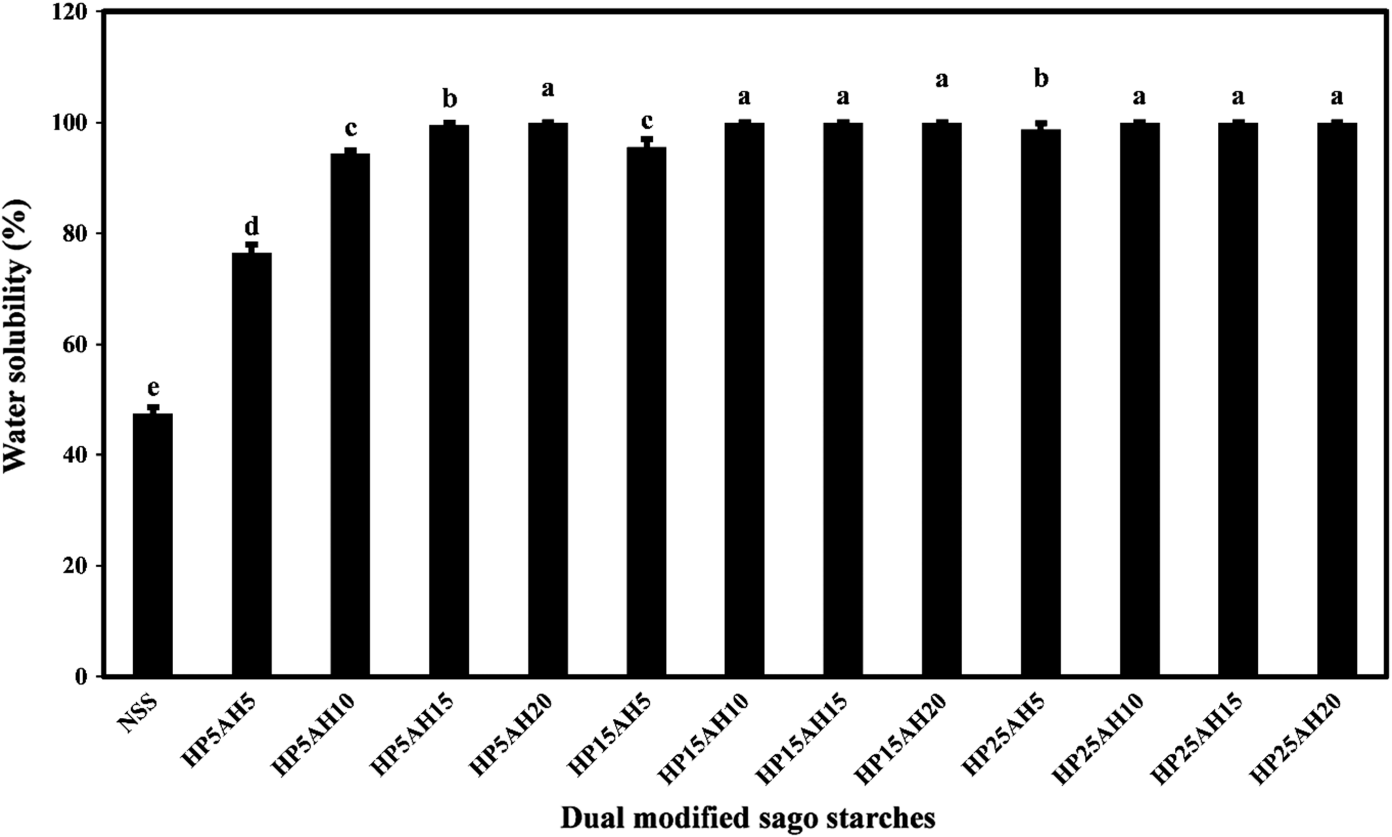
Comparison of water solubility values (%) of native and dually modified sago starch samples by acid hydrolysis‐hydroxypropylation. Bars represent mean (*n* = 3) ± *SD*. Different letters on the bars indicate a significant difference at 5% level of probability among starch samples. NSS, Native sago starch; the numbers after “HP” represent the propylene oxide ratio (%), and the numbers after “AH” represent the time (h) of acid hydrolysis

Figure 4 compared the average values of water solubility percentage of native and dual modified sago starch samples. The results of this test showed that dual modification of sago starch by acid hydrolysis‐hydroxylpropylation had a significant effect on increasing the water solubility of starches, and with increasing acid hydrolysis time and propylene oxide ratio, a significant increase in the solubility of starches was observed (*p* < .05), so that the water solubility value of native sago starch was 47.59%, and in HP5AH20, HP15AH10, HP15AH15, HP15AH20, HP25AH10, HP25AH15, and HP25AH20, the solubility reached 100%. Increasing water solubility of sago starch due to acid hydrolysis can be caused by breaking of amine and substructures into smaller structures as well as degradation of amylopectin structures and production of linear structures similar to amylose (Falsafi, 2019). During the acid modification of starch structure, H_3_O^+^ ions attack the glycosidic oxygen atom and hydrolyzed the glycosidic bonds. Therefore, acid preferentially attacks the amorphous region and increases the water solubility of hydrolyzed starch (Kaur et al., 2011). The increase in the water solubility of hydroxypropylated is due to the increase in the hydrophilicity of modified starch, which facilitates the penetration of water into the granules and improves the release of amylose from the amorphous regions of starch (Arueya & Ojesanmi, 2019). The introduction of bulky hydroxypropyl groups also weakens the granular structures of starch and facilitates their hydration (Gunaratne & Corke, 2007). The increased water solubility of various starches due to acid hydrolysis and hydroxypropylation process has also been observed by other researchers (Biduski et al., 2017; Hartiningsih et al., 2020; Koksel et al., 2008; Li et al., 2020; Wattanachant et al., 2003).

**FIGURE 5:**
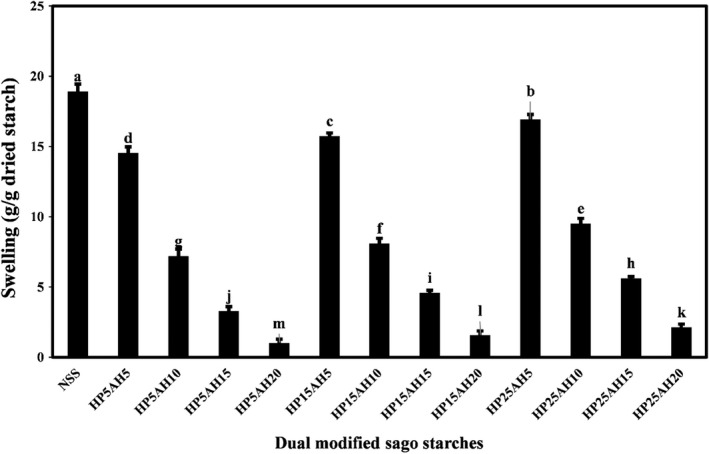
Comparison of swelling values (g/g dried starch) of native and dually modified sago starch samples by acid hydrolysis‐hydroxypropylation. Bars represent mean (*n* = 3) ± *SD*. Different letters on the bars indicate a significant difference at 5% level of probability among starch samples. NSS, Native sago starch; the numbers after “HP” represent the propylene oxide ratio (%), and the numbers after “AH” represent the time (h) of acid hydrolysis

One of the major structural features of starches is their ability to absorb water and swell in several stages until granule decomposition. The swelling capacity of starch indicates the interactions of the polymeric chains, including the crystalline and amorphous fractions (Krithika & Ratnamala, 2019). The mean values of swelling of native and dual modified sago starches by acid hydrolysis‐hydroxypropylation are compared in Figure 5. As can be seen in the figure, the highest rate of swelling was related to native sago starch (18.93 g/g dried starch), and dual modification of sago starch resulted in a significant reduction in starch swelling (*p* < .05). At constant levels of propylene oxide, with increasing the acid hydrolysis time from 5 to 20 h, the swelling rate of starches decreased significantly (*p* < .05). Decreased swelling of starches due to the acid hydrolysis process may be due to an increase in the high proportion of soluble dextrin in the short and long chains in the starch granules (John et al., 2002). However, at constant times of hydrolysis, with increasing propylene oxide ratio from 5% to 25%, the swelling of starches significantly increased (*p* < .05). This increase is probably due to the fact that during the hydroxypropylation process, the starch structure weakens and the water penetration into the granules increases, as a result of which the water absorption capacity of the starch granules increases and their swelling power increases (Singh et al., 2004). In general, the lowest swelling rate was observed in HP5AH20 (1.04 g/g dried starch). Similarly, Aparicio‐Saguilán et al. (2005), Falsafi (2019), Li et al. (2020), and Kaur et al. (2011) also observed a decrease in water absorption and swelling power of starches due to the acid hydrolysis process. The higher water absorption capacity of hydroxypropylated sweet potato, kudzu root, and barley starches compared to native starches was reported by Arueya and Ojesanmi (2019), Tang et al. (2012), and Devi and Sit (2019), respectively.

### 3.4 The pasting properties

Pasting properties are one of the most important characteristics of starch, especially in industrial applications, which show the behavior of starch during the heating process in the presence of sufficient amounts of water over time. In this experiment, the slurry of starch is subjected to a series of heating and cooling stages, and changes in its viscosity are recorded with time and temperature by a rapid viscosity analyzer (RVA) (Keskin et al., 2021). Table 1 shows the pasting properties of native and dual modified sago starches by acid hydrolysis‐hydroxypropylation. Dual modification of the sago starch structure resulted in a significant reduction of the pasting temperature, the time to reach the peak viscosity, peak viscosity, and final viscosity of the modified starches compared to native sago starch (*p* < .05). At constant levels of propylene oxide, with increasing acid hydrolysis time from 5 to 20 h, the pasting properties of sago starch decreased, and also at constant times of acid hydrolysis, increasing the ratio of propylene oxide from 5% to 25% led to a significant reduction in pasting properties of modified starches (*p* < .05). The pasting temperature, the time to reach the peak viscosity, peak viscosity, and final viscosity of the sago starch samples, were in the range of 56.57–73.11°C, 1.86–4.83 min, 236.55–414.38 cP, and 283–323.40 cP, respectively. In general, the highest amount of pasting properties was observed in the native sago starch, and the lowest amount of these properties was related to HP25AH20. The results obtained from the study of pasting properties of modified sago starch generally demonstrated an increase in the strength of intermolecular bonding forces due to the chemical modification of the starch structure. The decrease in the peak and final viscosity of sago starch due to hydroxypropylation is due to the fact that hydrophilic groups weaken the network and cause excess water to enter the starch micelle and increase the solubility of starch (Lawal, 2009); therefore, reaching the peak viscosity occurs at a lower time and temperature. The acid hydrolysis process improved the hydroxypropylation process of starch and increased the reaction between hydroxypropyl groups and molecular compounds of starch, and thus, increasing the acid hydrolysis time and propylene oxide ratio reducing the viscosity in starch pastes. Research has shown that chemical modification of starch structure by acid hydrolysis reduces the molecular weight of starch and increases the number of free aldehyde groups, thereby reducing the viscosity and increasing the solubility of starch (Thys et al., 2013). These results were consistent with those obtained by Olayinka et al. (2015), Surendra Babu et al. (2015), and Tang et al. (2018). Basilio‐ Cortés et al. (2019) also agreed that the application of acid hydrolysis pretreatment before the succinylation process of corn starch caused more degradation of the starch granules than the use of succinylation alone and further reduced the peak viscosity of the dual modified starch pastes.

**TABLE 1:** Pasting properties of the native and dual modified sago starches

Starches	Pasting temperature (°C)	Time to reach peak viscosity (min)	Peak viscosity (cP)	Final viscosity (cP)
NSS	73.11 ± 0.36a	4.83 ± 0.09a	414.38 ± 4.06a	323.40 ± 1.27a
HP5AH5	68.76 ± 0.17b	3.44 ± 0.13b	391.23 ± 3.89b	306.87 ± 1.40b
HP5AH10	66.98 ± 0.29c	3.21 ± 0.06c	384.47 ± 2.91c	305.90 ± 0.70b
HP5AH15	65.73 ± 0.20d	3.00 ± 0.14d	378.91 ± 1.87d	303.29 ± 1.63c
HP5AH20	64.62 ± 0.34e	2.76 ± 0.05e	374.90 ± 2.29d	302.59 ± 1.32c
HP15AH5	64.37 ± 0.21e	2.54 ± 0.11f	362.46 ± 1.80e	298.60 ± 1.78d
HP15AH10	63.13 ± 0.29f	2.25 ± 0.10g	359.18 ± 1.71e	296.22 ± 1.73de
HP15AH15	62.12 ± 0.21g	2.04 ± 0.06h	354.08 ± 1.58f	294.26 ± 1.28ef
HP15AH20	60.89 ± 0.17h	1.91 ± 0.06i	351.09 ± 1.39g	292.35 ± 1.27fg
HP25AH5	58.38 ± 0.20i	2.28 ± 0.07g	346.09 ± 2.38h	290.12 ± 0.94gh
HP25AH10	57.51 ± 0.26j	2.13 ± 0.09gh	344.08 ± 1.15h	288.10 ± 1.51h
HP25AH15	57.10 ± 0.21j	2.01 ± 0.07hi	340.75 ± 3.14i	285.07 ± 1.12i
HP25AH20	56.57 ± 0.15k	1.86 ± 0.06i	336.55 ± 2.09i	283.32 ± 1.70i

Note

The values are mean ±SE (*n* = 3). Different letters show significant differences at 5% level of probability between values in the same columns. NSS, Native sago starch; the numbers after “HP” represent the propylene oxide ratio (%), and the numbers after “AH” represent the time (hours) of acid hydrolysis.

### 3.5 The gelatinization properties and glass transition temperature

During heating of starch granules in the presence of excess water, crystalline structures, which are mainly composed of amylopectin branched structures, are converted into amorphous sections during an endothermic process. The energy required to convert crystal structures of starch into amorphous parts is determined by a differential calorimetric test. Gelatinization temperature refers to the temperature at which the crystal structures of the starch granules gradually melt to an amorphous form. Table 2 shows the thermal properties of native and dual modified sago starches by acid hydrolysis‐hydroxypropylation. The highest amount of To, Tp, Tc, and ΔH was observed in native sago starch, and dual modification of starch structure by acid hydrolysis‐hydroxypropylation significantly reduced the gelatinization temperatures and gelatinization enthalpy of modified starches compared to native starch (*p* < .05). At a constant ratio of propylene oxide, increasing the acid hydrolysis time from 5 to 20 h partially increased the gelatinization temperatures and decreased the enthalpy of modified starch samples, but these changes were not significant in most cases. The slight decrease in enthalpy of acid hydrolyzed starches is probably due to the fact that acid hydrolysis destroys some of the double helixes in both the crystalline and amorphous regions, thereby melting fewer double helixes during gelatinization and the enthalpy slightly reduced (Wang & Wang, 2001). However, at constant acid hydrolysis time, by increasing the propylene oxide ratio from 5% to 25%, the temperatures and enthalpy of gelatinization significantly decreased (*p* < .05). During the hydroxypropylation process, the active groups introduced into the starch chains are able to destroy intramolecular and intermolecular bonds and increase water access and decrease gelatinization temperature. The decrease in gelatinization enthalpy due to hydroxypropylation is also related to the degradation of double helixes in amorphous regions by hydroxypropyl groups. As a result, the number of double helixes that dissolve and melt during gelatinization will be lower in hydroxypropylated starches. The values of the To, Tp, Tc, and ΔH of different sago starch samples were in the range of 63.40–70.82°C, 72.77–78.92°C, 75.27–82.11°C, and 10.44–13.38 J/g, respectively. Gelatinization enthalpy indicates the energy required to destroy the organized structure of starch. In fact, low enthalpy demonstrates less stability of starch crystals and the structural order (Oh et al., 2019). Most studies on the modification of starch structures with hydroxypropylation process reported a decrease in the temperatures and enthalpy of gelatinization (Javadian et al., 2021; Mehboob et al., 2021; Oladebeye et al., 2019; Park & Kim, 2020; Shaikh et al., 2017; Wang & Shi, 2020). Tang et al. (2012) also observed a decrease in gelatinization temperatures and enthalpy of acid‐modified Kudzu root starch by hydroxypropylation process. No effect of acid hydrolysis on the enthalpy of starch gelatinization was reported by some researchers (Biduski et al., 2017; John et al., 2002).

**TABLE 2:** The gelatinization properties of the native and dual modified sago starches

Starches	To (°C)	Tp (°C)	Tc (°C)	ΔH (J/g)
NSS	70.82 ± 0.30a	78.92 ± 0.19a	82.11 ± 0.11a	13.38 ± 0.13a
HP5AH5	68.82 ± 0.12b	77.21 ± 0.10c	79.86 ± 0.12d	12.40 ± 0.08b
HP5AH10	68.90 ± 0.18b	77.35 ± 0.11c	80.05 ± 0.15cd	12.22 ± 0.11b
HP5AH15	68.96 ± 0.17b	77.43 ± 0.14bc	80.26 ± 0.11bc	11.96 ± 0.14c
HP5AH20	69.05 ± 0.19b	77.58 ± 0.10b	80.36 ± 0.13b	11.92 ± 0.09c
HP15AH5	65.90 ± 0.13c	75.75 ± 0.13d	77.08 ± 0.11g	11.50 ± 0.11d
HP15AH10	65.99 ± 0.12c	75.75 ± 0.12d	77.22 ± 0.15fg	11.48 ± 0.10d
HP15AH15	66.15 ± 0.21c	75.94 ± 0.16d	77.42 ± 0.14ef	11.31 ± 0.08de
HP15AH20	66.20 ± 0.17c	76.00 ± 0.21d	77.65 ± 0.12e	11.19 ± 0.08e
HP25AH5	63.40 ± 0.12e	72.82 ± 0.12e	75.27 ± 0.14i	10.85 ± 0.08f
HP25AH10	63.57 ± 0.18de	72.84 ± 0.13e	75.42 ± 0.13hi	10.64 ± 0.12g
HP25AH15	63.72 ± 0.14d	73.05 ± 0.20e	75.49 ± 0.16hi	10.56 ± 0.08g
HP25AH20	63.85 ± 0.12d	72.77 ± 0.27e	75.68 ± 0.14h	10.44 ± 0.10g

Note

The values are mean ±SE (*n* = 3). Different letters show significant differences at 5% level of probability between values in the same columns. NSS, Native sago starch; the numbers after “HP’ represent the propylene oxide ratio (%), and the numbers after “AH” represent the time (h) of acid hydrolysis.

Abbreviations: TC, completion temperature; TO, onset temperature; TP, peak temperature; ΔH, enthalpy.

**FIGURE 6:**
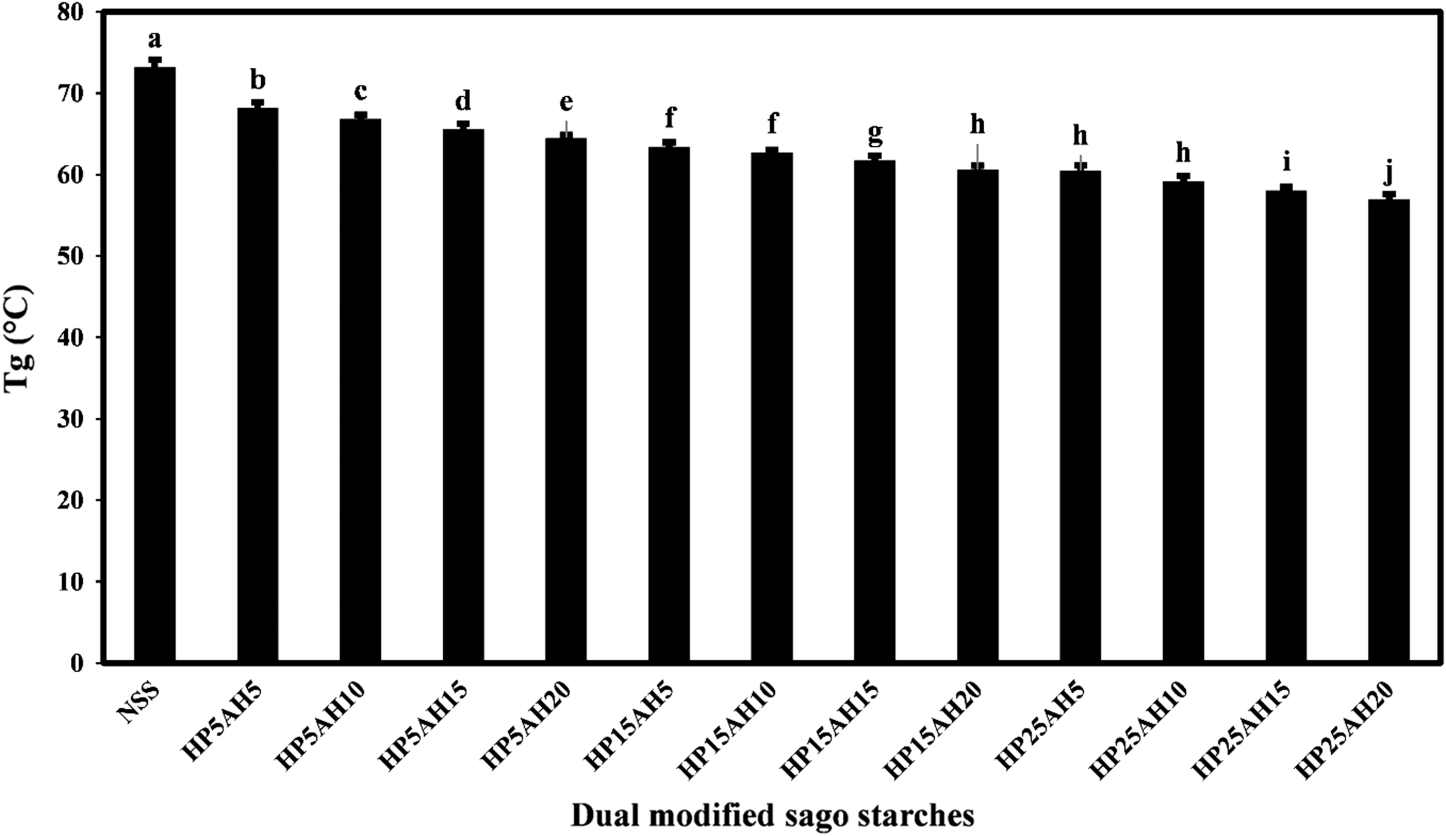
Comparison of Tg values (°C) of native and dually modified sago starch samples by acid hydrolysis‐hydroxypropylation. Bars represent mean (*n* = 3) ± *SD*. Different letters on the bars indicate a significant difference at 5% level of probability among starch samples. NSS, Native sago starch; the numbers after “HP” represent the propylene oxide ratio (%), and the numbers after “AH” represent the time (h) of acid hydrolysis

The glass transition temperature (Tg) is the temperature at which the glass state becomes a rubbery state. The more crystalline and glassy the starch polymer and the more orderly it is, the higher the Tg (Oh et al., 2019). The Tg values of native and dual modified sago starches by acid hydrolysis‐hydroxypropylation are compared in Figure 6. The native sago starch had the highest Tg (73.25°C), and the dual modification of starch led to a significant reduction in this temperature (*p* < .05). Increasing the propylene oxide ratio and acid hydrolysis time caused a significant decrease in Tg (*p* < .05). The lowest Tg was observed in HP25AH20 (57.01°C). In general, acid hydrolysis and hydroxypropylation reduced the order of sago starch structure and thus significantly reduced the Tg compared to native sago starch (*p* < .05). Decreased Tg of modified starches due to acid hydrolysis and hydroxypropylation process has been reported by other researchers (Chatakanonda et al., 2011; Chotipratoom et al., 2015; Omojola et al., 2010; Yeh & Yeh, 1993).

### 3.6 The retrogradation of starch

Changes in starch during gelatinization and retrogradation are important to evaluate its functional properties for food processing, during digestion and in industrial applications (Chang et al., 2021). These characteristics evaluate the acceptance quality, shelf life, and nutritional value of the final products (Wang & Copeland, 2013). Retrogradation of starch often has adverse effects because it participates in the process of staling bread and other starch‐rich foods, reducing the shelf life and acceptance of products and producing a lot of waste. When starch is heated in the presence of water and then cooled, the amylose and amylopectin chains are broken down into new structures called starch retrogradation (Hoover et al., 2010). Table 3 presents the retrogradation properties of native and dual modified sago starches by acid hydrolysis‐hydroxypropylation. As can be seen in the table, the highest temperatures and enthalpy of retrogradation were observed in the native sago starch, and dual modification of starch structure led to a significant reduction in retrogradation compared to the native starch (*p* < .05). At constant acid hydrolysis time, by increasing the propylene oxide ratio from 5% to 25%, the temperatures and enthalpy of retrogradation significantly decreased (*p* < .05). At a constant ratio of propylene oxide, increasing the time of acid hydrolysis also caused a slight decrease in starch retrogradation temperatures, but these reductions weren't significant in most cases. The lowest temperatures and enthalpy of retrogradation were obtained in the HP25AH20. The To, Tp, Tc, and ΔH of retrogradation of sago starch samples were in the range of 44.16–54.18°C, 54.74–60.27°C, 58.99–65.25°C, and 1.05–5.16 J/g, respectively.

**TABLE 3:** The retrogradation properties of the native and dual modified sago starches

Starches	To (°C)	Tp (°C)	Tc (°C)	ΔH (J/g)
NSS	54.18 ± 0.19a	60.27 ± 0.13a	65.25 ± 0.32a	5.16 ± 0.12a
HP5AH5	51.25 ± 0.11b	59.00 ± 0.20b	62.61 ± 0.14b	3.69 ± 0.10b
HP5AH10	51.14 ± 0.10bc	58.93 ± 0.19b	62.58 ± 0.13b	3.57 ± 0.11c
HP5AH15	50.97 ± 0.16c	58.76 ± 0.14b	62.53 ± 0.10b	3.49 ± 0.13d
HP5AH20	50.95 ± 0.15c	58.64 ± 0.16b	62.44 ± 0.13b	3.50 ± 0.09e
HP15AH5	47.46 ± 0.16d	56.83 ± 0.14c	61.09 ± 0.19c	2.49 ± 0.16f
HP15AH10	47.47 ± 0.14d	56.69 ± 0.17c	60.95 ± 0.21c	2.33 ± 0.09f
HP15AH15	47.26 ± 0.12d	56.66 ± 0.14c	60.81 ± 0.23c	2.19 ± 0.10g
HP15AH20	47.17 ± 0.19d	56.54 ± 0.15c	60.77 ± 0.20c	2.18 ± 0.12h
HP25AH5	44.60 ± 0.18e	55.17 ± 0.12d	59.38 ± 0.13d	1.17 ± 0.13h
HP25AH10	44.49 ± 0.11e	54.99 ± 0.17de	59.27 ± 0.18de	1.13 ± 0.15h
HP25AH15	44.39 ± 0.20ef	54.88 ± 0.19de	59.27 ± 0.12de	1.11 ± 0.08i
HP25AH20	44.16 ± 0.21f	54.74 ± 0.15e	58.99 ± 0.23e	1.05 ± 0.10j

Note

The values are mean ±SE (*n* = 3). Different letters show significant differences at 5% level of probability between values in the same columns. NSS: Native sago starch; the numbers after “HP” represent the propylene oxide ratio (%), and the numbers after “AH” represent the time (h) of acid hydrolysis.

Abbreviations: TC, completion temperature; TO, onset temperature; TP, peak temperature; ΔH, enthalpy.

In the process of hydroxypropylation, with the entry of hydroxypropyl groups into the starch chains, the destruction of intermolecular and intramolecular bonds occurs, and thus the starch structure is weakened. As a result, the mobility of the starch chains in the amorphous regions increases. In this way, the retrogradation of starch is reduced (Olayinka et al., 2015). Since there are two different pathways for the effect of the acid hydrolysis process on starch retrogradation, the effects of this process are different under various conditions. On the one hand, the removal of α (1→6) branching points in the amylopectin and amylose hydrolysis occurs due to the acid hydrolysis, which can increase the retrogradation intensity of starch gels. On the other hand, the small molecules in the residual acidic hydrolyzed starch have an irregular effect on the recrystallization of the hydrolyzed starch gels and demonstrate the opposite effect (Wang et al., 2015). Similarly, previous research has shown that the hydroxypropylation of different starches reduced the tendency of starch samples to retrograde (Chotipratoom et al., 2015; Lawal et al., 2008; Oh et al., 2019; Senanayake et al., 2014). Kang et al. (1997) found that due to acid hydrolysis and increasing the time of this process, retrogradation of rice starch increased. However, Gunaratne and Corke (2007) reported that acid hydrolysis had no significant effect on the retrogradation of corn and potato starches but reduced the intensity of retrogradation in wheat starch. There are generally conflicting reports on the effect of the acid hydrolysis process on the rate of starch retrogradation (Wang et al., 2015).

### 3.7 Syneresis

The stability of starch pastes can be shown by their syneresis values. Syneresis indicates the amount of water that separates from the starch granules after the freezing‐thawing cycle or during the storage period (Wang et al., 2015). Higher values of syneresis indicate less stability of starch gel and a higher rate of starch retrogradation (Liu et al., 2014). Indirectly, the retrogradation and syneresis properties of starches are influenced by the structural arrangement of starch chains in the amorphous and crystalline regions of the starch granules (Kaur et al., 2007).

**FIGURE 7:**
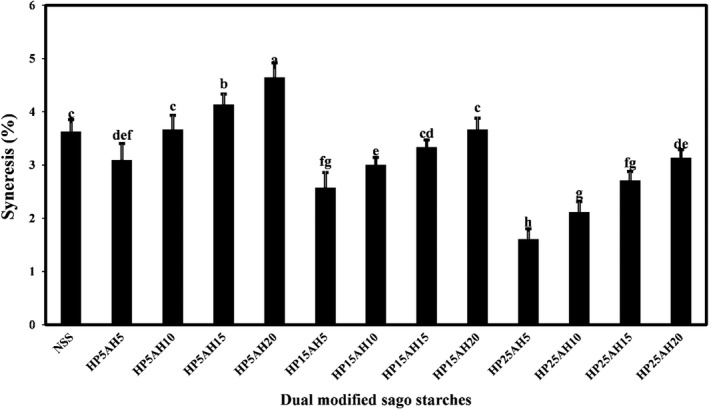
Comparison of syneresis values (%) of native and dually modified sago starch samples by acid hydrolysis‐hydroxypropylation. Bars represent mean (*n* = 3) ± *SD*. Different letters on the bars indicate a significant difference at 5% level of probability among starch samples. NSS, Native sago starch; the numbers after “HP” represent the propylene oxide ratio (%), and the numbers after “AH” represent the time (h) of acid hydrolysis

The syneresis percentage of native and dual modified sago starches by acid hydrolysis‐hydroxypropylation is compared in Figure 7, which shows that at constant acid hydrolysis time, by increasing the propylene oxide ratio from 5% to 25%, the syneresis value of modified starch significantly decreased (*p* < .05). However, at a constant ratio of propylene oxide, increasing the acid hydrolysis time from 5 to 20 h led to a significant increase in the percentage of syneresis (*p* < .05). The highest and lowest syneresis values were observed in HP5AH20 (4.65%) and HP25AH5 (1.61%), respectively. The syneresis is due to rearrangement and subsequent crosslinking between the amylopectin and amylose, but because the hydroxypropyl groups attached to the amylopectin and amylose during the hydroxypropylation process create a spatial barrier, this barrier prevents the chains from coming too close to each other during storage and reduces syneresis (Kaur et al., 2007). The hydrophilic hydroxypropyl groups can increase the water holding capacity of starch molecules and reduce syneresis in modified starches. The higher syneresis of starch samples due to the increase in acid hydrolysis time is probably due to the reduction of water holding capacity and water absorption of acid hydrolyzed starches compared to the native starch. A remarkable effect of hydroxypropylation process in reducing the syneresis tendency of various starches has been reported in several scientific studies (Kaur et al., 2007; Lawal et al., 2008; Lee & Yoo, 2011; Maulani et al., 2019; Senanayake et al., 2014; Shaikh et al., 2017). Increased syneresis of sweet potato, banana, and wheat starches gels due to the acid hydrolysis process has also been observed by Kaur et al. (2011).

## 4 CONCLUSION

In this study, it is observed that the dual modification of sago starch structure by acid hydrolysis‐hydroxypropylation improves the starch water solubility. Dual modification of starches at low acid hydrolysis time and high ratio of propylene oxide led to a remarkable reduction in the retrogradation intensity and syneresis percentage of modified starches compared to native sago starch. Due to the dual modification, the structure of sago starch became more irregular, and the peak viscosity occurred in less time. The results of this study generally demonstrated that the dual modified sago starch, especially low acid hydrolysis time and high ratio of propylene oxide, can be used in the food industry to reduce the staling rate and syneresis of food products.

## ACKNOWLEDGEMENTS

We thank Prof. Fazilah Ariffin from Universiti Sains Malaysia for her valuable input and critical reading of this manuscript.

## CONFLICT OF INTEREST

The authors declare no conflict of interest.

## ETHICAL APPROVAL

This study does not involve any human or animal testing.

## Data Availability

The data that support the findings of this study are available from the corresponding author, upon reasonable request.
